# Regiospecific Profiles of Fatty Acids in Triacylglycerols and Phospholipids from Adzuki Beans (*Vigna angularis*)

**DOI:** 10.3390/nu20100049

**Published:** 2010-01-19

**Authors:** Hiromi Yoshida, Yuka Tomiyama, Naoko Yoshida, Kyoko Shibata, Yoshiyuki Mizushina

**Affiliations:** 1 Department of Nutritional Sciences, Kobe Gakuin University, Kobe, Hyogo, 651-2180 Japan; Email: tomiyama@nutr.kobegakuin.ac.jp (Y.T.); s-mail.kobegakuin.ac.jp (K.S.); mizushin@nutr.kobegakuin.ac.jp (Y.M.); 2 Department of Biomedical Sciences, College of Life Sciences, Ritsumeikan University, Kusatsu, Shiga 525-8577 Japan; Email: nyoshida@sk.ritsumei.ac.jp; 3 Cooperative Research Center of Life Sciences, Kobe Gakuin University, Kobe, Hyogo, 650-8586 Japan

**Keywords:** adzuki beans (*Vigna angularis*), fatty acids, phosphatidylcholine, phosphatidyl- ethanolamine, phosphatidylinositol, regiospecific characteristics, triacylglycerols

## Abstract

Regiospecific distributions of fatty acids (FA) of triacylglycerols (TAG) and phospholipids (PL) isolated from five cultivars of adzuki beans (*Vigna angularis*) were investigated. The lipids comprised mainly PL (72.2-73.4 wt-%) and TAG (20.6-21.9 wt-%), whilst other components were detected in minor proportions (0.1-3.4 wt-%). The principal profiles of the FA distribution in the TAG and PL were evident in the beans among the five cultivars: unsaturated FA were predominantly distributed in the *sn*-2 position, whilst saturated FA primarily occupied the *sn*-1 or the *sn*-3 position in the these lipids. The results would be useful information to both producers and consumers for manufacturing traditional adzuki confectionaries such as *wagashi* in Japan.

## 1. Introduction

Adzuki or small red beans (*Vigna angularis*) are a popular ingredient in many confections in the Orient. The predominant use of adzuki in traditional Japanese confections is a paste or *wagashi* such as *youkan*, *manju* and *amanatto* [1,2,3]. Adzuki beans are a rich source of carbohydrates, protein, vitamins, minerals and fiber [4]; however, they also contain antinutritional factors. Phytates, α-galactosides and trypsin inhibitors are among these factors, and their concentrations differ widely among the different cultivars of adzuki beans. Therefore, when adzuki beans are used for confectionaries, they are boiled in a cooker and yield a hot water extract as a by-product, which is known to contain active ingredients, but is discarded. It has been reported that the 40% (w/v) ethanol fraction of the hot-water extract from adzuki beans suppresses not only proliferation of human stomach cancer cells in culture but also benzo(α)pyrene-induced tumorigenesis in the mouse forestomach [5]. Thus, the hot-water extract of adzuki beans has a number of effects [6,7]. Wu *et al.* [8] have shown recently that a water-soluble extract of the adzuki beans could inhibit acetaminophen-induced liver damage. Han *et al.* [9] have reported the protective action of an adzuki extract against acetaminophen-induced hepatotoxicity *via* a hepatic γ-glutamylcysteinylglycine (GSH)-mediated antioxidation/detoxification system in rat liver after four weeks of feeding. 

In the Western world, because of the potential effect of saturated fatty acids (FA) on atherosclerosis, coronary heart disease, and myocardial infarction, the consumption of fat is gradually shifting from food products containing animal fats to food products containing vegetable oils. Legume lipids and dietary fibers are being suggested for dietary reduction of blood cholesterol because legumes contain substantial amounts of desirable polyunsaturated fatty acids (PUFA) and fiber [10]. The nutritive value of adzuki beans has been investigated in studies concerned with the chemical composition of the whole seed reported by Hayakawa and Breene [11], Tjahjadi *et al.* [4], Hsieh, Pomeranz and Swanson [12]. Knowledge of TAG structure has also become increasingly important since the stereospecific structure influences the lipid metabolism [13] and bioavailability of FA. However, little information is available about the dietary fiber content and FA composition of adzuki beans. Recently, we reported the lipid components, FA compositions and TAG molecular species of adzuki beans [14,15].

To the best of our knowledge, no data are available on the regiospecific profiles of the FA in the TAG and major PL such as phosphatidylethanolamine (PE), phosphatidylcholine (PC) and phosphatidylinositol (PI). The objectives of the present investigation were to study the differences in the composition and regiospecific profiles of FA in the TAG and PL isolated from adzuki beans of the five cultivars, in an attempt to evaluate the composition and quality characteristics of the lipids. The data obtained would be useful to both consumers and producers for manufacturing traditional confectioneries (*youkan, manju and amanatto*).

## 2. Results and Discussion

### 2.1. Lipid Components in the Beans

The major chemical components were as follows: moisture 3.5-3.7 wt-%, fat 1.3-1.5 wt-% and protein 20.3-21.5 wt-%. There were no significant differences (*P* > 0.05) in these contents among the five cultivars. Adzuki beans are high in complex carbohydrates, protein, and fiber, yet are extremely low in fat [16]. Predominant components were PL (72.2-73.4 wt-%) and TAG (20.6-21.9 wt-%), followed by steryl esters (SE: 2.4-3.3 wt-%), accompanied by very small amounts (2.4-3.7 wt-%) of other lipid components (data not shown). The adzuki beans are not oil seeds but typical vegetable seeds [17]. Therefore, the PL content is quite significant, whilst glycolipids are present only in trace amounts, indicating that PL form the principal components of the cell membranes in the beans. 

The profiles of major PL components of the adzuki beans are represented in Table 1. The original amounts of each PL were approximately 4489-5641 mg (45.4-46.9 wt-%), 2492-2947 mg (24.6-25.8 wt-%), 2304-2945 mg (23.3-24.7 wt-%) and 544-589 mg (4.8-5.7 wt-%) per 1.0 kg of beans for PC, PI, PE and others, respectively. Others include diphosphatidylglycerol, phosphatidic acid and phosphatidylglycerol. It is generally known that these PL are the essential components of the cell membranes in plant. Because membrane lipids are involved in such fundamental cell processes as ion transport, energy generation and biological reactions, they are highly conserved in terms of both quality and quantity [18].

**Table 1:** The content of major phospholipids in the oils obtained from adzuki beans^†^

Cultivar	Phosphatidylethanolamine				Phosphatidylcholine				Phosphatidylinositol				Others			
	(mg/kg beans)														
Toyomi	2304	±	103^a^		4489	±	203^a^		2552	±	123^a^		544	±	23^a^		
	-23.3				-45.4				-25.8				-5.5		
Akane	2380	±	105^b^		4680	±	215^a^		2492	±	118^a^		577	±	25^bc ^		
	-23.5				-46.2				-24.6				-5.7		
Roman	2850	±	132^d^		5641	±	253^c^		2947	±	128^b^		589	±	27^c^		
	-23.7				-46.9				-24.5				-4.9		
Otome	2945	±	138^c^		5462	±	231^b,c^		2946	±	124^b^		572	±	23^b,c^		
	-24.7				-45.8				-24.7				-4.8		
Erimo	2709	±	128^c^		5304	±	218^b^		2811	±	120^b^		557	±	18^a,b^		
	-23.8				-46.6				-24.7				-4.9		

^†^Mean values ± standard error. Each value is the average of three replicates, and is expressed as mg/kg beans. Values in parentheses are relative wt-% contents of the individual lipids in phospholipids. "Others" include minor phospholipids components such as diphosphatidylglycerol, phosphatidic acid and phosphatidylglycerol. Values in the same column with different superscripts are significantly different from those of the individual cultivars (*P* < 0.05).

### 2.2. FA Composition of total Lipids and PL

The FA compositions of total lipids and PL in the beans were compared among the five cultivars (data not shown). The principal FA components are generally palmitic (16:0), stearic (18:0), oleic (18:1n-9), linoleic (18:2n-6) and α-linolenic (18:3n-3) acids, the distribution of which varies according to these lipid classes. Moreover, long-chain saturated FA (20:0 and 22:0) were also detected at low percentages (0.8-3.2 wt-%) in these lipids. These samples had large amounts of total unsaturated FA which consisted mainly of linoleic (18:2n-6) acid, followed by α-linolenic (18:3n-3) and oleic (18:1n-9) acids, representing 70.6-73.8 wt-% and 69.9-72.6 wt-% for total lipids and PL, respectively. Adzuki beans have a low fat content, which is, however, of good quality because of high levels of unsaturated FA in most of them (Table 2 and Table 3).

Some significant differences (*P <* 0.05) in FA compositions were found when comparing total lipids and PL. With a few exceptions, the percentage of palmitic (16:0) acid was higher (*P* < 0.05) in the PL (25.8-26.5 wt-%), whilst α−linolenic (18:3n-3) acids was higher (*P* < 0.05) in the total lipids (22.5-24.3 wt-%) or TAG (25.4-27.4 wt-%) as shown in Table 2. However, the percentage of linoleic (18:2n-6) acid was less (*P* < 0.05) in the TAG (31.3-32.7 wt-%) than that in the total lipids (45.1-47.4 wt-%) or PL (47.3-48.3 wt-%). These profiles in the FA distributions are not similar to the results reported in typical vegetable seeds such as pea seeds [19] or kidney beans [20]. 

### 2.3. FA positional Distribution of TAG

The composition and positional distribution of FA in the TAG were compared among the three cultivars *Toyomi, Roman* and *Erimo* (Table 2). The major components were palmitic (16:0), linoleic (18:2n-6) and α-linolenic (18:3n-3) acids, followed by oleic (18:1n-9), stearic (18:0), arachidic (20:0) and behenic (22:0) acids. Unsaturated FA (>96%), such as linoleic (18:2n*-*6) and α-linolenic (18:3n-3), were predominantly concentrated in the *sn*-2 position of the TAG molecules, whilst saturated FA, such as palmitic (16:0), stearic (18:0), arachidic (20:0) and behenic (22:0), were primarily located in the *sn*-1 or the *sn*-3 position. However, oleic (18:1n-9) acid was almost evenly distributed in the *sn-*1, 2, or 3 position, as other researchers have also reported [21]. The data for *Akane* and *Otome* were omitted from Table 2 because these profiles were very similar to the results for *Toyomi, Roman* and *Erimo. *No significant differences (*P *> 0.05) existed in the FA distributions for TAG among the five cultivars. These trends were essentially the same as those obtained from other cultivars as previously reported [14,22]. Therefore, the regiospecific distribution patterns in the FA of TAG were very similar to the results obtained for other seed lipids such as soybeans and corn [23].

**Table 2:** Composition and positional distribution of fatty acids in the triacylglycerols isolated from adzuki beans^†^.

Cultivar	Position	Fatty acid (wt-%)																	
		16:00			18:00			18:01			18:02			18:03			Others		
Toyomi	Total	24.8	±	1.0^a^	3.8	±	0.1^a^	7.4	±	0.3^a^	32	±	1.2^a^	27.4	±	1.1^b^	4.6	±	0.1^a^
	*sn*-2	2.1	±	0.1^b^	------			6.5	±	0.2^a^	57.8	±	2.3^a^	33.6	±	1.3^b^	------		
	*sn*-1.3	36.2	±	1.5^a^	5.7	±	0.2^b^	7.9	±	0.3^c^	19.1	±	0.6^a^	27.8	±	1.0^b^	3.3	±	0.2^a^
Roman	Total	25.3	±	1.1^a^	4.2	±	0.1^c^	7.6	±	0.3^a^	31.9	±	1.3^a^	25.4	±	1.4^a^	5.6	±	0.1^b^
	*sn*-2	3.1	±	0.1^c^	------			9.3	±	0.4^b^	57.7	±	2.3^a^	29.9	±	1.3^a^	------		
	*sn*-1.3	36.4	±	1.5^a^	6.3	±	0.1^b^	6.8	±	0.2^a^	20.5	±	0.9^b^	23.2	±	1.0^a^	6.8	±	0.2^a^
Erimo	Total	25	±	1.0^b^	3.7	±	0.1^a^	7.3	±	0.3^a^	31.9	±	1.3^b^	26.8	±	1.3^a,b^	5.3	±	0.1^b^
	*sn*-2	2.2	±	0.1^b^	0.6	±	0.1^a^	9.5	±	0.4^b^	57.3	±	2.3^a^	30.4	±	1.3^a^	------		
	*sn*-1.3	36.4	±	1.3^a^	5.3	±	0.1^a^	6.2	±	0.1^a^	19.8	±	0.8^a^	25	±	1.3^b^	7.3	±	0.2^b^

^†^Mean values ± standard error. Each value represents the average of three replicates, and is expressed relative wt-% contents of the individual fatty acids. "Others" include minor fatty acids such as 14:0, 16:1, 20:0 and 22:0. Values in the same column with different superscript are significantly different from those of the individual cultivars (*P* < 0.05)

### 2.4. FA Positional Distribution of Major PL

As shown in Table 3, the composition and positional distributions of FA in the PE, PC and PI were compared between the two cultivars *Akane* and *Otome*. However, the data for *Toyomi, Roman* and *Erimo *were omitted from Table 3 because their distribution patterns were very similar to the results for *Akane* and *Otome*.

**Table 3:** Composition and positional distribution of fatty acids of major phospholipids obtained from adzuki beans^†^.

Phospho-lipid	Cultivar	Position	Fatty acid (wt-%)																					
			16:00			18:00				18:01			18:02				18:03				Others			
Phosphatidyl ethanolamine	Akane	Total	35.2	±	1.5^a^		2.5	±	0.1^b^	5.3	±	0.2^a^		44.8	±	2.0^a^		11.6	±	0.5^a^		0.6	±	0.1^a^	
		*sn*-2	46.8	±	2.0^b^		2.8	±	0.1^a^	3.9	±	0.2^a^		35.8	±	1.6^a^		9.8	±	0.3^a^		0.9	±	0.1^a^	
		*sn*-1.3	23.8	±	1.1^a^		1.8	±	0.1^a^	6.7	±	0.2^c^		54	±	2.3^a^		13.4	±	0.6^a^		0.3	±	0.1^b^	
	Otome	Total	34.3	±	1.3^a^		2.3	±	0.1^a^	5.4	±	0.2^a^		44.7	±	2.0^a^		12.5	±	0.6^b^		0.8	±	0.1^b^	
		*sn*-2	43	±	2.0^a^		2.5	±	0.1^a^	5	±	0.2^b^		36.6	±	1.7^a^		11.6	±	0.5^b^		1.3	±	0.0^c^	
		*sn*-1.3	25.6	±	1.2^b^		2.1	±	0.1^b^	5.8	±	0.2^a^		52.8	±	2.4^a^		13.4	±	0.6^a^		0.3	±	0.1^b^	
Phosphatidyl choline	Akane	Total	23.9	±	1.1^a^		3.3	±	0.1^b^	6.5	±	0.2^b^		49.9	±	2.1^a^		14.8	±	0.5^a^		1.6	±	0.1^a^	
		*sn*-2	31.6	±	1.4^b^		4.9	±	0.1^a^	6.7	±	0.2^c^		40.5	±	2.0^a^		13.6	±	0.3^a^		2.7	±	0.2^c^	
		*sn*-1.3	16.2	±	0.6^a,b^		1.7	±	0.1^a^	6.3	±	0.2^a^		59.3	±	2.4^a^		16	±	0.6^a^		0.5	±	0.1^a^	
	Otome	Total	24	±	1.0^b,c^		2.8	±	0.1^a^	6.8	±	0.2^b^		50	±	2.1^a^		14.9	±	0.5^a^		1.5	±	0.5^a^	
		*sn*-2	30.7	±	1.3^b^		3.6	±	0.1^b^	7.6	±	0.3^b^		41.8	±	2.0^a,b^		13.9	±	0.3^a^		2.4	±	0.3^a^	
		*sn*-1.3	17.3	±	0.6^b^		2	±	0.1^b^	6	±	0.2^a^		58.2	±	2.4^a^		15.8	±	0.6^a^		0.5	±	0.6^a^	
Phosphatidyl inositol	Akane	Total	43.4	±	2.0^a^		3.4	±	0.1^a^	4.8	±	0.1^a^		32.6	±	1.5^a,b^		15	±	0.5^b^		0.8	±	0.1^a^	
		*sn*-2	52	±	2.3^b^		4.7	±	0.1^a^	3.6	±	0.1^a^		26.7	±	1.2^a,b^		12.6	±	0.4^b^		0.4	±	0.1^a^	
		*sn*-1.3	34.8	±	1.5^a^		2.1	±	0.1^a^	6	±	0.2^b^		38.5	±	1.5^a^		17.4	±	0.6^b^		1.2	±	0.1^b^	
	Otome	Total	43.2	±	2.0^a^		3.6	±	0.1^b^	5	±	0.2^a^		32.5	±	1.8^a,b^		14.8	±	1.0^a^		0.9	±	0.1^a^	
		*sn*-2	50.4	±	2.3^a^		4.7	±	0.1^a^	3.7	±	0.3^b^		26.7	±	1.6^a,b^		13.7	±	1.0^b^		0.8	±	0.1^c^	
		*sn*-1.3	36	±	1.5^b^		2.5	±	0.1^c^	6.3	±	0.3^b^		38.3	±	1.8^a^		15.9	±	1.0^a^		1	±	0.1^a^	

^†^Mean values ± standard error. Each value represents the average of three replicates, and is expressed as wt-% contents of the individual fatty acids. "Others" include minor fatty acids such as 14:0, 16:1, 20:0 and 22:0. Values in the same column with different superscripts are significantly different from those of the individual cultivars (*P* < 0.05).

The major FA in the three PL were commonly palmitic (16:0), stearic (18:0), oleic (18:1n-9), linoleic (18:2n-6) and α-linolenic (18:3n-3) acids. These FA distributions were very similar to each other in the individual PL among the five cultivars. However, significant differences (*P* < 0.05) in these FA distributions for PC (Table 3) were observed among the five cultivars. When comparing the three PL among the five cultivars, the percentage of linoleic (18:2n-6) acid was significantly (*P *< 0.05) higher in PC (49.5-50.8 wt-%) than that in PE (44.7-45.3 wt-%), whilst the percentage of palmitic (16:0) was significantly (*P* < 0.05) higher in PE (34.3-35.3 wt-%) than that in PC (22.8-24.6 wt-%), respectively. Furthermore, PI was unique in that it had the highest saturated FA content (47-48 wt-%) among the three PL (Table 3), although their distribution patterns were very similar among the five cultivars. Particularly, the percentage of palmitic (16:0) acid was significantly (*P* < 0.05) higher in PI (43.2-44.5 wt-%) than in PE or PC among the five cultivars. The regiospecific distributions of FA were very similar to each other in the major three PL among the five cultivars (Table 3). Generally, these distribution of FA in PE, PC, and PI was observed in all PL: saturated FA were mostly located in the *sn*-1 position (34.6-58.7 wt-%), whilst unsaturated FA predominantly occupied the *sn*-2 position (60.1-81.9 wt-%) of these molecules. With a few exceptions, these trends were essentially the same as those obtained from other cultivars as previously reported [22]. However, these results obtained are some differences in the positional distributions of FA for the PE, PC and PI of the other plant seeds reported in the literature [19,24].

## 3. Experimental Section

### 3.1. Adzuki Beans

The mature adzuki beans (*V. angularis*) used in this study were from the five different Japanese cultivars-*Erimo, Otome, Roman, Akane* and *Toyomi*-harvested at Tokachi, Hokkaido in Japan during the summer of 2008 that were furnished from Hokkaido Tokachi Area Regional Food Processing Technology Center. These beans were selected for uniformity based on bean weight of 112-153 mg for *Eriomo* and *Otome,* 128-165 mg for *Roman,* 170-216 mg for *Akane* and 225-262 mg for *Toyomi,* respectively. The beans were hand-selected to eliminate those that were cracked or otherwise damaged. Beans of each cultivar were divided into groups and stored in separate stainless steel containers at -25 °C until used.

### 3.2. Reagents and Standards

All chemicals and solvents used were commercially available extra-pure-grade products (Nacalai Tesque, Kyoto, Japan), but diethyl ether was further purified to remove peroxides according to the method described previously [25]. Thin-layer chromatography (TLC) pre-coated silica gel 60 plates (20 × 20 cm, 0.25 mm thickness) were purchased from Merck (Darmstadt, Germany). A TLC standard mixture containing monoacylglycerols (MAG), diacylglycerols (DAG), free fatty acids (FFA), TAG, SE and hydrocarbons (HC), was purchased from Nacalai Tesque. A PL kit from Serdary Research Laboratory (Mississauga, ON, Canada) was used as PL standard. 

Lipase from porcine pancreas was used after purification with previously described methods [25]. Phospholipase A_2_ was from bee (*Apis mellifera*) venom. Both enzymes (lipase and Phospholipase A_2_) were purchased from Sigma Chemical Co. (St. Louis, MO, USA). FA methyl ester (FAME) standards (F & OR mixture #3) were from Altech-Applied Science (State College, PA, USA). Methyl pentadecanoate (C15:0, 100 mg; Merck) was dissolved in *n*-hexane (20 mL) and used as the internal standard. Boron trifluoride (BF_3_) in methanol (14%; Wako Pure Chemical, Osaka, Japan) was used to prepare FA methyl esters (FAME). 

### 3.3. Chemical Analysis

The AOAC [26] methods were used to determine the chemical composition of the beans. Samples were analyzed in replicate for fat, protein and moisture contents according to the standard methods. Fat content was determined by solvent extraction (Method 991.36), protein content by a Kjeldahl method (Method 981.10) and moisture content by oven-drying to constant weight at 105 °C (Method 925.40). 

### 3.4. Extraction of Lipids

These beans (500 seeds) were ground to pass through a 0.5 mm sieve, using a Maxim homogenizer (Nihonseiki Kaisha, Ltd., Tokyo, Japan) at high speed for 10 min at 0 °C with 300 mL of chloro-form/methanol (2:1, vol/vol) fortified with 0.01% BHT, which was added to inhibit the oxidative degradation of lipids during analysis. The homogenate was vacuum-filtered through defatted filter paper on a Buchner funnel, and the filter residue was rehomogenized with a second volume of chloroform/methanol. The filtrates were combined and concentrated in a rotary vacuum evaporator at 35 °C. The residue was dissolved in 100 mL of chloroform/methanol (2:1, vol/vol), then 20 mL aqueous potassium chloride (0.75 wt-%) was added [27], and the phases were vigorously mixed. After phase separation, the chloroform layer was withdrawn, dried over anhydrous Na_2_SO_4_ and filtered, and the filtrate was concentrated under vacuum with a rotary evaporator at 35 °C. The extracted lipids were weighed to determine the lipid content of the beans and then carefully transferred to a brown-glass volumetric flask (25-mL) with chloroform/methanol (2:1, vol/vol).

### 3.5. Lipid Analysis

Using previously reported methods [28], total lipids were separated into eight fractions by preparative TLC on silica gel 60. Bands corresponding to HC, SE, TAG, FFA, 1,3-DAG, 1,2-DAG, MAG and PL were scraped off into test-tubes [105 × 16 mm; poly (tetrafluoroethylene)-coated screw caps]. Methyl pentadecanoate (10-100 mg) from a standard solution (5 mg/mL) was added to each tube as the internal standard with a microsyringe (Hamilton, Reno, NV, USA). With the exception of HC, FAME were prepared from the isolated lipids by heating with silica-gel for 30 min at 80 °C in BF_3_/methanol on an aluminium block bath [29]. Following a previously reported method [18], the *n-*hexane layer containing the FAME was recovered and dried over anhydrous Na_2_SO_4_. The solvent was then vaporized under a gentle stream of nitrogen, and the residue was analyzed by Shimadzu Model-14B GC (Shimadzu, Kyoto, Japan) equipped with a hydrogen flame ionization detector and a polar capillary column (ULBO HE-SS-10 for FAME fused silica WCOT [serial no. PSC 5481], cyanopropyl silicone, 30 m x 0.32 mm i.d.; Shinwa Chem. Ind., Ltd., Kyoto, Japan).

The oven temperature was programmed from an initial temperature of 180 °C (2 min hold), rising to 200 °C at a rate (2 °C /min), and held for 15 min. Helium was used as a carrier gas at a flow rate (1.5 mL/min), and the GC was operated under a constant pressure of 180 kPa. All samples were dissolved *n-*hexane for injection. The injection port and the flame ionization detector were maintained at 250 °C. Identification was made by comparison of retention times to those of standard FAME. The detection limit was 0.05 wt-% of total FA for each FAME in a FAME mixture, and the results are expressed as wt-% of total FAME.

Samples of the extracted polar lipids, obtained as described above, were further separated by TLC into several fractions with chloroform/methanol/acetic acid/deionized water (170:30:20:7, by volume) as the mobile phase. PL classes were detected by iodine vapor and were consistent with the authentic standards. Bands corresponding to PE, PC, PI and others were carefully scraped off into test tubes. Then, FAME were prepared by the same method as described above and analyzed by GC.

### 3.6. Enzymatic Hydrolysis of Lipids

TAG hydrolysis *invitro* was carried out according to the method reported previously [25]. The purified TAG (10 mg) were hydrolyzed with pancreatic lipase (15 mg) at 37 °C in 0.25 M Tris buffer (pH 7.5, 5 mL) containing 0.01 M CaCl_2_ (0.1 mL) and 0.1 wt-% deoxylcholate (0.25 mL) in a test tube (10-mL). A time period of 30 min was selected, based on the results of preliminary experiments using the standard TAG (glyceryl-1,3-myristate-*sn*-2-oleate: Sigma Chemical Co.). After approximately 60% of the TAG was hydrolyzed, adding of 6 M HCl (0.5 mL) and ethanol (1 mL) stopped the reaction. In that study, no FA (oleic acid) in the *sn*-2 position of standard TAG is transferred to the *sn*-1 or the *sn*-3 position within 60% hydrolysis (for 30 min). The reaction products were separated by TLC as already described [25]. The FFA and *sn*-2 MAG bands were carefully scraped off the plate and then methylated [29]. The procedure was checked by comparing the FA compositions of the original TAG and the TAG remaining after partial hydrolysis. 

The regiospecific profile of FA in each of the PE, PC and PI samples isolated by preparative TLC was determined by phospholipase A_2_ hydrolysis [30]. Briefly, each PL (3-5 mg) was suspended in 0.25 M Tris buffer (pH 7.5, 0.5 mL) containing 0.01 M CaCl_2_ in a Erlenmeyer flask (10 mL). To this suspension, phospholipase A_2 _(5 mg) and diethyl ether (10 mL) were added. The reaction mixture was incubated at 28 °C for up to 15 h with continuous shaking under a nitrogen atmosphere. The hydrolysis was almost complete (>98%) in this period, as judged from a preliminary experiment using the standard PL (L-3-PC, 2-oleoyl-1-palmitoyl-*sn*-glycero-3-PC; Sigma Chemical Co.). Diethyl ether was evaporated under nitrogen, and samples were extracted with chloroform/methanol (2:1, vol/vol). To obtain the different products after hydrolysis, the lipid extracts were subjected to one-dimensional TLC with chloroform/methanol/deionized water (65:20:2, by volume), respectively. The spots were visualized with iodine vapor, and the bands corresponding to the FFA and the lyso-PL were separately scraped off into the text tubes. The constituent FA were analyzed by GC after transmethylation as described above.

### 3.7. Statistical Analysis

All preparations and determination were carried out in replicate, and the results were subjected to one-way analysis of variance (ANOVA) [31]. Significant differences (*P *< 0.05) were calculated using multiple comparison test, following a previously reported method [32].

## 4. Conclusions

The regiospecific profile of FA in TAG and PL isolated from adzuki beans (*Vigna angularis*) was compared among five cultivars. The principal profiles of FA distributions in the TAG evident in adzuki beans were: unsaturated FA, especially linoleic (18:2n-6) acid, were predominantly concentrated in the *sn*-2 position, whilst saturated FA, especially palmitic (16:0) and stearic (18:0) acids, primarily occupied the *sn*-1 position or the *sn*-3 position in the lipids. The regiospecific profile of FA in PE, PC and PI was observed in all PL: unsaturated FA predominantly occupied the *sn*-2 position. The lipid components and FA distributions were almost the same among the five cultivars. The five cultivars in this study contained linoleic (18:2n-6) and α-linolenic (18:3n-3) acids as their dominant FA. The low total lipid contents of adzuki beans is similar to other foods (cereals as rice and rye, and legumes as bean, chick-pea and beans) that have less than 2% of fat, but with the adavantage that the fat material of the bean possesses a suitable PUFA n-6/n-3 relation of high effect in human and animal nutrition. Therefore, these regiospecific profiles of FA in TAG and major PL might be not influenced by genetic variability and planting location as previously described [14,15,22]. 

## Acknowledgments

We are grateful to Kiyoshi Oba (Hokkaido Tokachi Area Regional Food Processing Technol- ogy Center) for kindly providing us with adzuki beans. We also thank Bruce Holub of the Department of Human Health and Nutritional Sciences, University of Guelph, Canada, for reviewing and commenting on this manuscript. This work was supported by a Grant-in-Aid for Scientific Research (C) no. 20500730 (HY) from Japan Society for the Promotion of Science (JSPS), a Grant-in-Aid for Kobe-Gakuin University Joint Research (A) and “Life Science Center for Cooperative Research” Project for Private Universities: matching fund subsidy from MEXT. 
